# Transcriptomic Analysis of the Mussel *Elliptio complanata* Identifies Candidate Stress-Response Genes and an Abundance of Novel or Noncoding Transcripts

**DOI:** 10.1371/journal.pone.0112420

**Published:** 2014-11-06

**Authors:** Robert S. Cornman, Laura S. Robertson, Heather Galbraith, Carrie Blakeslee

**Affiliations:** 1 Leetown Science Center, United States Geological Survey, Kearneysville, West Virginia, United States of America; 2 Northern Appalachian Research Branch (Leetown Science Center), United States Geological Survey, Wellsboro, Pennsylvania, United States of America; Glasgow Caledonian University, United Kingdom

## Abstract

Mussels are useful indicator species of environmental stress and degradation, and the global decline in freshwater mussel diversity and abundance is of conservation concern. *Elliptio complanata* is a common freshwater mussel of eastern North America that can serve both as an indicator and as an experimental model for understanding mussel physiology and genetics. To support genetic components of these research goals, we assembled transcriptome contigs from Illumina paired-end reads. Despite efforts to collapse similar contigs, the final assembly was in excess of 136,000 contigs with an N50 of 982 bp. Even so, comparisons to the CEGMA database of conserved eukaryotic genes indicated that ∼20% of genes remain unrepresented. However, numerous candidate stress-response genes were present, and we identified lineage-specific patterns of diversification among molluscs for cytochrome P450 detoxification genes and two saccharide-modifying enzymes: 1,3 beta-galactosyltransferase and fucosyltransferase. Less than a quarter of contigs had protein-level similarity based on modest BLAST and Hmmer3 statistical thresholds. These results add comparative genomic resources for molluscs and suggest a wealth of novel proteins and noncoding transcripts.

## Introduction

Environmental degradation, including anthropogenic stressors such as habitat alterations, contaminants, and fluctuations in physico-chemical parameters (e.g., particulates, flow, salinity, and temperature), are aggravating the ongoing, rapid, and global decline of freshwater mussel biodiversity [1]. Because of their response to a wide range of stressors, marine and freshwater mussels are frequently used as early indicators of environmental insults that may have ecosystem-level ramifications [2]. Researchers are increasingly exploiting gene-expression analysis to study both the mechanisms of stress response and the range of conditions eliciting those reactions, as well as to identify sensitive, early-onset, nonlethal assays of stress agents applicable to natural populations. Here we have undertaken a transcriptome analysis of the mussel *Elliptio complanata*, widespread in eastern North America, to support mechanistic studies of aquatic toxicology and to aid the discovery of exposure biomarkers. We annotate transcripts homologous to genes from other molluscs and model organisms (fruit fly and nematode), and identify several gene-family expansions within the *E. complanata* lineage. Unexpectedly, we find that a large fraction of transcript space in *E. complanata* is novel or noncoding, highlighting the need for more functional genomic research in bivalves.

## Methods

### Specimen collection and RNA extraction

*E. complanata* were collected by hand from the mainstem Delaware River in Wayne County, PA (near Callicoon, NY; GPS coordinates 41.7671062N -75.0659450W) and transported to the USGS Northern Appalachian Research Branch in Wellsboro, PA. Collections were made under State of Pennsylvania Scientific Collector's Permit number 467 Type II and did not involve endangered or protected species. Mussels were originally housed at the ambient water temperature of the collection site, but were gradually acclimated to a stable laboratory temperature of 20°C by raising the temperature 2°C per 24-hour period. Sampled mussels were part of an RNA-Seq experiment assessing transcriptional response to salt exposure, the results of which will be reported elsewhere. Four sampled mussels were maintained at 2 ppt NaCl for seven days (the treatment condition) while an additional four mussels experienced ∼0.1 ppt NaCl (the control condition).

After the experimental period, the eight mussels were gently pried open and a small clipping of mantle tissue removed, following [3], and preserved in RNAlater (Applied Biosystems/Life Technologies). Total RNA was later extracted from mantle tissue using the RNeasy Fibrous Tissue Mini Kit (Qiagen), following manufacturer's instructions and including an on-column DNase treatment. RNA integrity was evaluated for a subset of samples using a 2100 BioAnalyzer (Agilent Technologies). However, because invertebrates have a break in the 28S rRNA, no RIN was computed. Instead, size profiles were evaluated by eye for consistency. For each of the eight mantle tissue samples, 5 µg total RNA was sent to Eurofins MWG Operon for library preparation and sequencing. mRNA was enriched by polyA capture. Multiplexed libraries were run together in a single Illumina HiSeq 2000 flow cell.

### Transcriptome assembly

All bioinformatics programs were implemented with default analysis parameters unless otherwise indicated. Read pairs were first screened for adapter sequence and compositional bias using CLC Genomics Workbench v.5 (Qiagen) and FastQC [4]. Based on that analysis, we trimmed the first 14 bp of all reads to ensure removal of exogenous sequence. We then trimmed based on quality score with CLC Genomics Workbench using a minimum phred-equivalent quality score of 20 and a minimum length of 50, with at most two ambiguous bases allowed.

Reads passing quality filtering, including broken pairs, were assembled in CLC Genomics Workbench using automatic selection of the “bubble size” parameter, which modulates the handling of polymorphsim- or repeat-associated bubbles in the DeBruijn graph. Distance bounds for read pairs were set to a minimum of 150 bp and a maximum of 350 bp, based on fragment length data provided by the sequence provider. The mean inter-pair distance of assembled read pairs was 243 bp.

After assembly with CLC Genomics Workbench, we performed a secondary clustering of contigs with cd-hit-est v. 4.5.4 [5] using default settings. We then searched for potential chimeras by identifying contigs with BLASTX (v. 2.2.21) hits to two different predicted proteins (with an e-value less than 1e-20), separated by at least 100 bp. BLASTX searches were against the oyster *Crassostrea gigas* [6], another mollusc that was generally the highest scoring taxon in GenBank against our assembly. Contigs that had a smaller gap (<100 bp) between the two distinct BLASTX matches were evaluated by eye using the graphical output of the NCBI Blast web server, supplemented with searches against *Drosophila melanogaster* as well. In total, 134 contigs were split on the midpoint between the two BLASTX matches and substituted with the 268 revised contigs. We next purged contigs that were MegaBLAST matches to a longer contig at 90% identity over half their length. Finally, we evaluated whether CAP3 (version date 12/21/07) [7] run on the assembled contigs would substantially reduce the assembly size, using an overlap of 100 bases at 90% identity. This step produced a negligible reduction in the assembly size and therefore was not retained. A more aggressive alternative was also evaluated, in which each set of contigs that matched by MegaBlast was submitted to CAP3 assembly under loose parameters: 40 bp overlap at 85% identity. After re-clustering the resulting contigs with cd-hit-est, the total number of contigs was reduced by only ∼500. We therefore concluded that the assembly was recalcitrant to further reductions in contig number and excluded the CAP3 contigs because the limited gain was offset by the increased risk of producing erroneous assemblies. Remaining contigs less than 200 bp were excluded to conform with NCBI submission requirements for the TSA database. The data can be accessed under BioProject PRJNA194430.

### Transcriptome annotation

Contigs were searched by BLASTX against NCBI RefSeq protein models of *Drosophila melanogaster* and *Caenorhabditis elegans* and the molluscs *C. gigas* and *Lottia gigantea* [8], by TBLASTX against the extensive mRNA models available for the oyster *Crassostrea angulata* [9], and by TBLASTX against the *C. gigas* genome. Any contigs not matched against those data sets were searched by BLASTX against the UniRef100 protein sequences downloaded from UniProt [10]. For all contigs, ORFs were identified and coding potential assessed using PORTRAIT v. 1.1 [11]. Since the PORTRAIT score for coding potential is strand-specific, we retained the higher of the two scores for forward and reverse-complement sequences. Translated ORFs were searched for known protein domains of the Pfam-A database (downloaded 12/26/12) [12], using Hmmer3 [13] at an E-value threshold of 0.05. Kmer analysis was performed with jellyfish v. 1.1 [14]. Gene ontology analysis was based on the 9,827 *Drosophila* genes annotated with GO categories in the GO-Slim compilation [15] downloaded April 2013. Phylogenetic trees of gene families were computed with MEGA5 [16] using the maximum likelihood method and JTT protein substitution matrix.

## Results and Discussion

### Transcriptome assembly and annotation

Sequencing and assembly output is summarized in **Table 1**. We arrived at an initial assembly of 155,370 contigs > =  200 bp using CLC Genomics Workbench. After searching for chimeras, clustering with cd-hit-est, removing contigs with strong matches to other contigs by MegaBlast, and trimming contigs to conform to NCBI policy (see Materials and Methods), we retained 136,982 contigs. Of these, 22,088 were 1 kb or greater with the longest exceeding 16 kb (having similarity to an apolipoprotein gene of *D. melanogaster*, CG15828, which produces a 13-kb transcript). The assembly N50 was 982 bp and the mean GC content of transcripts was 35.3%.

**Table 1:** Sequencing and assembly statistics.

Feature	Value
Total reads in pairs	361,027,256
Paired and unpaired reads after quality filtering	304,853,114
Mean read length after quality filtering, bp	84.3
Fraction of reads used in initial assembly	74.6%
Contig number of final assembly	136,982
N50 of final assembly, bp	982
Number of contigs 1 kb or greater	22,088
Total assembly length, Mbp	88.9
GC content	35.3%

The large contig number and total assembly length (88.9 MB) initially suggested to us that a large amount of allelic sequence had been retained as distinct contigs. Several efforts to reduce the contig number through more lax clustering and secondary assembly methods had negligible success (see Methods). We also failed to detect untrimmed adapters using CLC Genomics or NCBI's recommended BLASTN settings for searching the UniVec database (www.ncbi.nlm.nih.gov/tools/vecscreen/univec/). All contigs passed NCBI's own contaminant screen during TSA submission, and we found no indication of contamination from other organisms: contig GC content had a broad but unimodal distribution (not shown) and BLAST results (see below) were consistent with the taxonomic position of *E. complanata*. A small number of microbial matches were spread amongst unrelated bacteria and viruses, had percent identities of 80% or less, and had modest E-values, suggesting that these were predominantly non-specific matches. Lacking a weight of evidence of contamination, we retained these contigs in the assembly.

**Figure 1:**
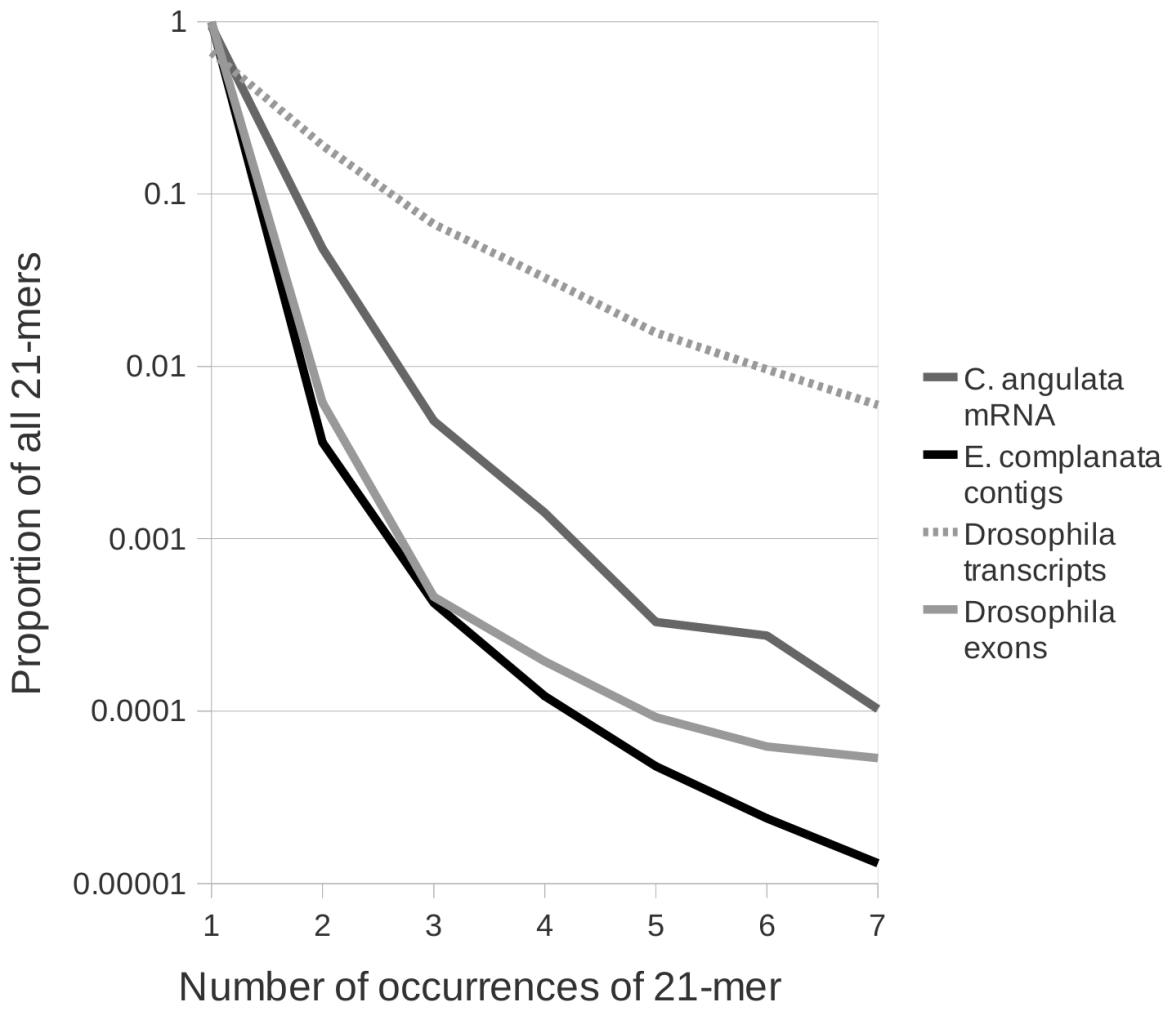
The proportion of unique sequence in the *Elliptio complanata* assembly and three other reference sequences. The total occurrences of all distinct 21-bp kmers was tabulated and the frequency of those occurring from one to seven times is shown on a log scale. The complete exome and transcriptome of *Drosophila melanogaster*, downloaded from BioMart [17], serve as lower and upper bounds of the redundancy of 21-mers that would be expected to be recovered from shotgun transcriptomics of that species. mRNA of *Crassostrea angulata* (Portuguese oyster) downloaded from GenBank and clustered at 90% identity serves as the most closely-related reference for molluscs currently available.

We next used kmer analysis to evaluate the level of redundancy that might be present in the assembly, either as assembly artifacts or genuine transcript variants. The proportion of all sequence words of arbitrary length *k* (“kmers”) that occur more than one time in a data set can be used as an index of sequence redundancy. We compared our *E. complanata* assembly with *D. melanogaster*, the closest high-quality model organism, and with the oysters *C. gigas* and *C. angulata*, which have extensive genome and transcriptome records in GenBank. We also included the *D. melanogaster* reference exon sequences from BioMart [17] in this comparison. We first used the same default cd-hit-est settings to separately cluster the *Drosophila* and *Crassostrea* sequences prior to comparison with *E. camplanata*. The proportion of unique 21-mers was highest in the *E. complanata* assembly and the proportion of 21-mers present multiple times was on par with the *D. melanogaster* exon set (**Figure 1**). The *E. complanata* assembly had substantially less sequence repetition than the *C. angulata* transcripts even after clustering with cd-hit-est had reduced the latter from 80,777 to 53,042 sequences. As expected, the *D. melanogaster* transcript set contained much more repetition than the other data sets because all known isoforms are included in the kmer counting, whereas a comparable shotgun sequencing effort would fail to recover many rare isoforms. Our rationale for including the *D. melanogaster* transcript set was to provide an approximate upper bound of kmer counts for a transcriptome rich in alternatively spliced isoforms. These kmer comparisons imply that our assembly does not contain unusual sequence repetition (which might arise due to allelic polymorphism, alternative splicing, or poorly performing assembly parameters), but instead appears to reflect a genuinely large number of expressed loci in *E. complanata*.

We used BLASTX against the CEGMA collection of conserved low-copy genes in eukaryotes [18] to assess the completeness of the transcriptome we recovered. We identified only 360 of the 458 proteins as potentially present even at a relatively relaxed E-value of 1e-15, suggesting the transcriptome is approximately 80% complete at the gene level, i.e. disregarding alternative transcripts. (Note that this comparison is not reciprocal, a common criterion for assessing orthology, because multiple transcript contigs may derive from a single locus.) While an incomplete capture of CEGMA genes is expected because only mantle tissue was represented in the sequencing libraries, a quantitative estimate of incompleteness remains useful for evaluating overall gene content and comparisons with other species. It further highlights the large transcriptome size of *E. complanata* given that a substantial portion of protein-coding genes remain undetected.

BLAST and hmmer results are summarized in **Table 1** and fully listed in **File S1.** On average there were slightly less than two *E. complanata* contigs matching each sequence of *C. gigas*, the mollusc *Lottia gigantean* [8], or *D. melanogaster* in **Table 2**. We infer this to be the rough upper-bound for the average number of transcript fragments deriving from the same *E. complanata* locus in the assembly, either as non-overlapping 5′ and 3′ primer regions or as alternative isoforms, although gene duplication after divergence of the *E. complanata* lineage may also contribute (see below).

**Table 2:** Number of unique BLAST matches between *Elliptio complanata* transcriptome contigs and each reference database.

Reference	*E. camplanata* contigs with matches	Proportion of matched sequences in reference genome*
*Crassostrea gigas* genome	20706	not applicable
*Crassostrea gigas* proteins	20433	10602/27873
*Lottia gigantea* proteins	21356	11152/23877
*Crassostrea angulata* mRNA	11231	7232/unknown
*Drosophila melanogaster* proteins	12189	6522/13937
*Caenhorhabditis elegans* proteins	5637	1453/20541

*The numerator is the number of sequences of each reference set that were best matches of at least one *E. complanata* contig, and the denominator the number of sequences in the reference set.

We detected homologs of a range of genes potentially responsive to common environmental stressors. These include twelve cytochrome P450 genes (“cP450” hereafter; see below), which as a class respond to a broad spectrum of toxins or other injurious agents [19]. Contig 790 encodes a copper-zinc superoxide dismutase, an important antioxidant [20]. Numerous contigs had significant similarity to heat-shock proteins, which protect against acute heat stress and have been implicated in the competitive advantage of an invasive saltwater mussel in warmer waters [21]. The alpha (Contig 32662) and beta (Contig 10867) subunits of a sodium/potassium transporting ATPase were identified, a homolog of which is responsive to salinity in fish [22]. Two short contigs (Contig 50876 and Contig 116297) were homologous to vitellogenin, a biomarker of female reproduction [23]. Another short contig, Contig 99924, encodes an aryl hydrocarbon receptor homolog, a receptor class that participates in the detoxification of aromatic hydrocarbons in vertebrates (see [24]). Contig 7881 encodes a member of the estrogen receptor gene family, but it is more similar to the orphan estrogen-related receptors (i.e., those having unknown endogenous ligands) and therefore presumably does not bind estrogens. No other estrogen receptor was found, but numerous neuroendocrine receptor homologs were identified (see **File S1**) that are expected to bind serotonin, dopamine, or acetylcholine based on BLAST matches and Pfam-A domains. While not intended to be a complete list of gene classes related to aquatic toxins or stress, these results illustrate the utility of transcriptomics for quickly capturing species-specific homologs of candidate genes better-characterized in other organisms.

### Gene family expansions

**Figure 2:**
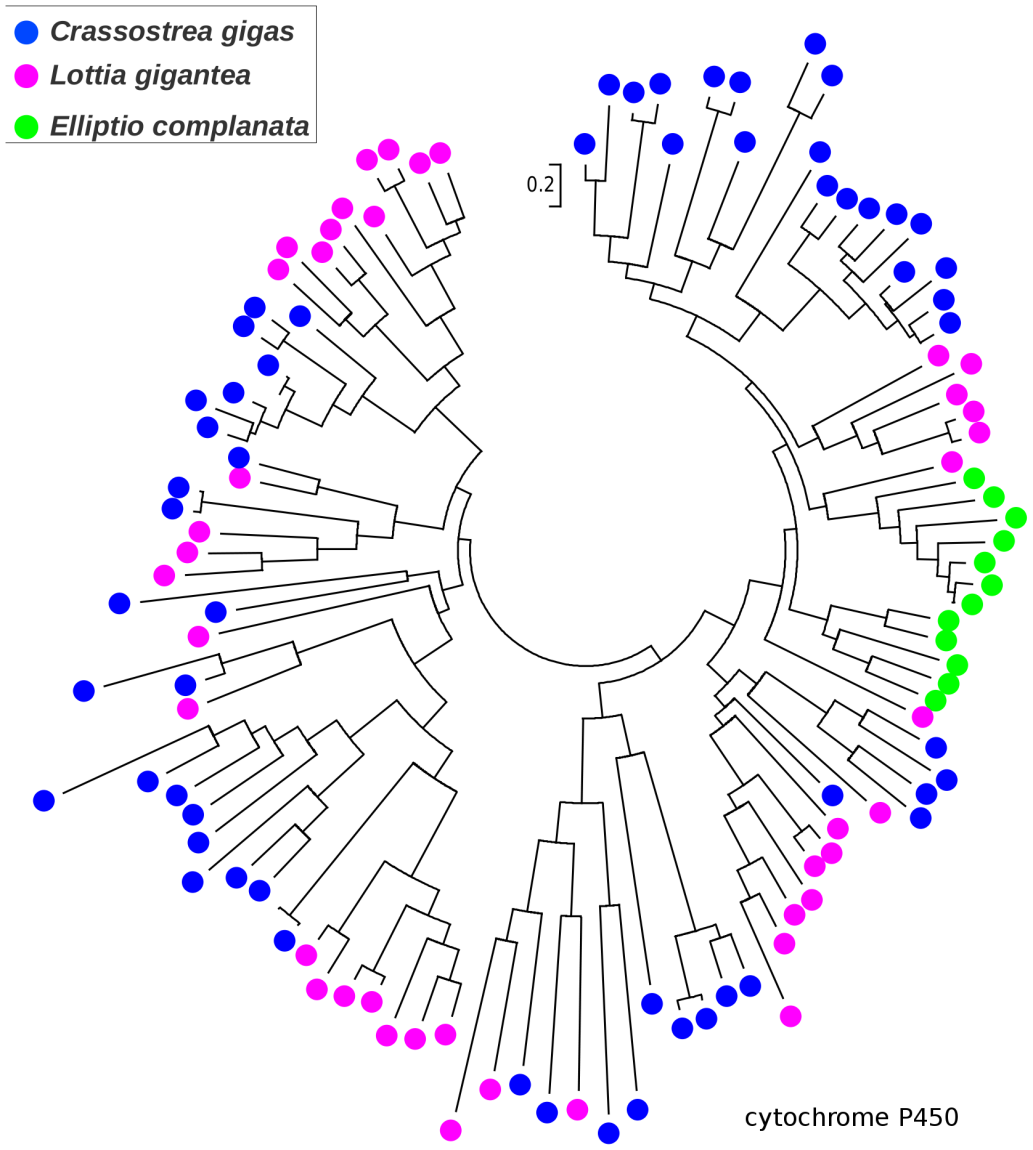
Gene tree of cytochrome P450 sequences from *Elliptio complanata* and two other molluscs suggesting lineage-specific diversification. Trees were rendered in MEGA5 [16] using maximum likelihood and the JTT amino-acid substitution matrix. Bootstrap support was not evaluated because no explicit phylogenetic hypothesis was tested. Multiple-sequence alignment with gene/contig names is provided in fasta format in the supplemental materials.

**Figure 3:**
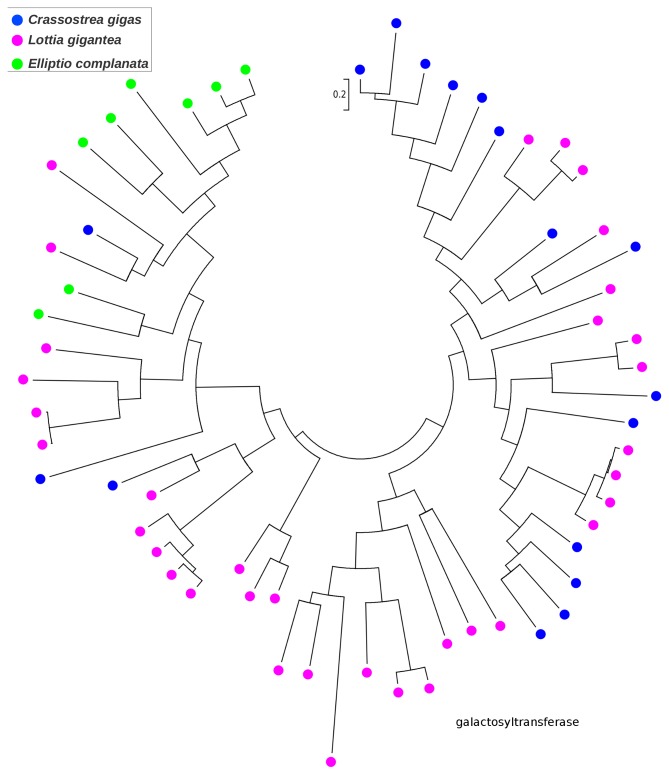
Gene tree of 1,3 beta-galactosyltransferase sequences from *Elliptio complanata* and two other molluscs suggesting lineage-specific diversification. Trees were rendered in MEGA5 [16] using maximum likelihood and the JTT amino-acid substitution matrix. Bootstrap support was not evaluated because no explicit phylogenetic hypothesis was tested. Multiple-sequence alignment with gene/contig names is provided in fasta format in the supplemental materials.

**Figure 4:**
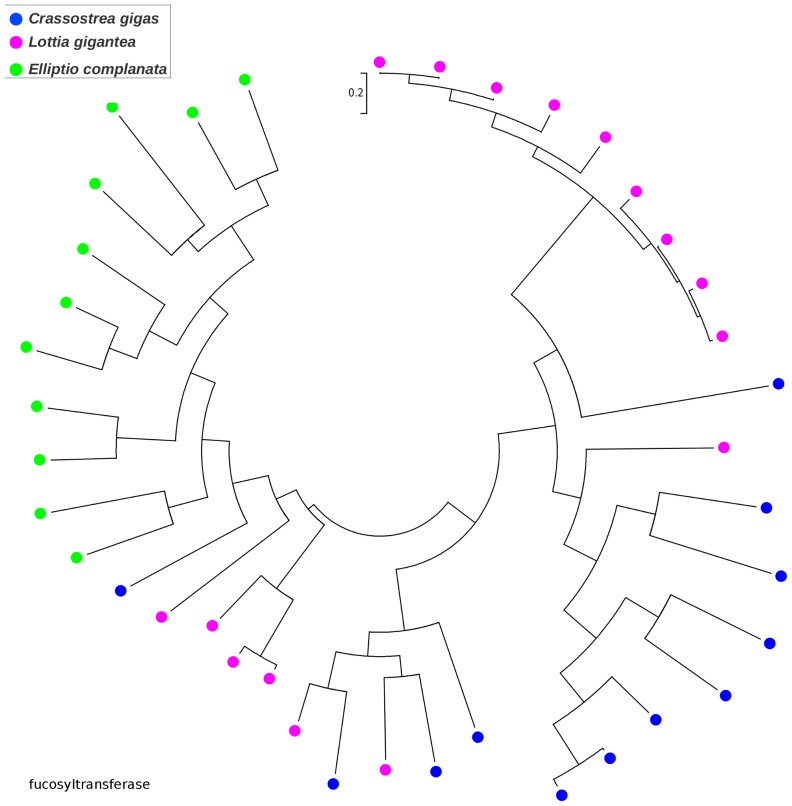
Gene tree of fucosyltransferase sequences from *Elliptio complanata* and two other molluscs suggesting lineage-specific diversification. Trees were rendered in MEGA5 [16] using maximum likelihood and the JTT amino-acid substitution matrix. Bootstrap support was not evaluated because no explicit phylogenetic hypothesis was tested. Multiple-sequence alignment with gene/contig names is provided in fasta format in the supplemental materials.

While most *C. gigas* proteins were the best BLASTX match of only one or a few *E. complanata* contigs, a small number were each the best matches of numerous *E. complanata* contigs. Hypothesized factors contributing to this skew include *E. complanata* genes that are weakly expressed, highly polymorphic, or that produce numerous splice variants, resulting in non-overlapping or uncollapsed allelic contigs. A chance abundance of high-scoring pairs from nonhomologous genes may also contribute. However, the pattern may also indicate gene duplication within the *E. complanata* lineage and/or gene loss within the *C. gigas* lineage since their divergence. Because BLAST matches alone do not provide an adequate picture of homology and evolutionary history (because high-scoring segment pairs are analyzed rather than phylogenetically informative multiple-sequence alignments), we used an ad-hoc phylogenetic approach to explore this latter possibility. We first identified the longest ORFs for clusters of ten or more *E. complanata* contigs with best matches to the same *C. gigas* protein and aligned these with Muscle [25]. Alignments were manually inspected for the presence of a broadly conserved domain constituting a large fraction of the alignment, and poorly aligned sequences were removed. For each of the 18 resulting alignments, we then downloaded all BLASTP matches (threshold expectation of e-20) to the recently sequenced *L. gigantea* [8], as well as to *C. gigas* (thus capturing paralogs of the original *C. gigas* gene that seeded the analysis), and recomputed the three-species multiple-sequence alignment with Muscle. We also required that all sequences in the alignment share a common Pfam domain. We then rendered phylogenetic trees in MEGA5 to identify cases of lineage-specific clustering (see Methods). The majority of initial seed alignments either produced poor multiple-sequence alignments or did not provide evidence of lineage-specific clustering. However, we did find extensive clustering by species for paralogs of the cP450 family (PF00067; **Figure 2**) and for two glycosyl transferase enzymes: beta 1,3 galactosyltransferase (PF01762; **Figure 3**) and alpha 1,3 fucosyltransferase (PF00852; **Figure 4**). Multiple sequence alignments underlying these gene trees, including gene/contig names, are included in **Files S2–S4**. The cP450 phylogeny is particularly interesting because of the role of these genes in xenobiotic metabolism and stress response, as noted above, and the rather limited number and diversity of *E. complanata* cP450s identified. The glycosyl transferases may contribute to the diversity of glycoproteins found in mussels, as components of shell or other extracellular matrices such as mucous. Of course, only genes expressed at an appreciable level could be captured by our transcriptome assembly, but it is implausible that the observed gene-tree topologies are simply an expression artifact with no contribution of lineage-specific evolution.

### Gene ontologies mapped from Drosophila

We investigated the ontologies of genes in *Drosophila* for which we could detect protein-sequence similarity in *E. complanata*, to identify patterns of conservation of biological function between the two species. We counted the number of occurrences of each GO-Slim term [15] associated with matched *Drosophila* homologs and compared them to counts for unmatched *Drosophila* genes. For each GO category associated with 20 or more genes in *D. melanogaster*, we graphed the proportion of those genes that were matched by at least one *E. complanata* transcript contig. This assessment of functional conservation necessarily reflects both homolog retention and rates of protein sequence divergence, and there are likely to be correlates such as gene length, expression level, and fraction of low-complexity sequence that bias the apparent conservation of genes in different ontological categories. Despite these limitations, the proportion of each GO category assigned to the 5,122 matched homologs of the 9,827 total genes with annotations provides a first view of functional divergence.

**Figure 5:**
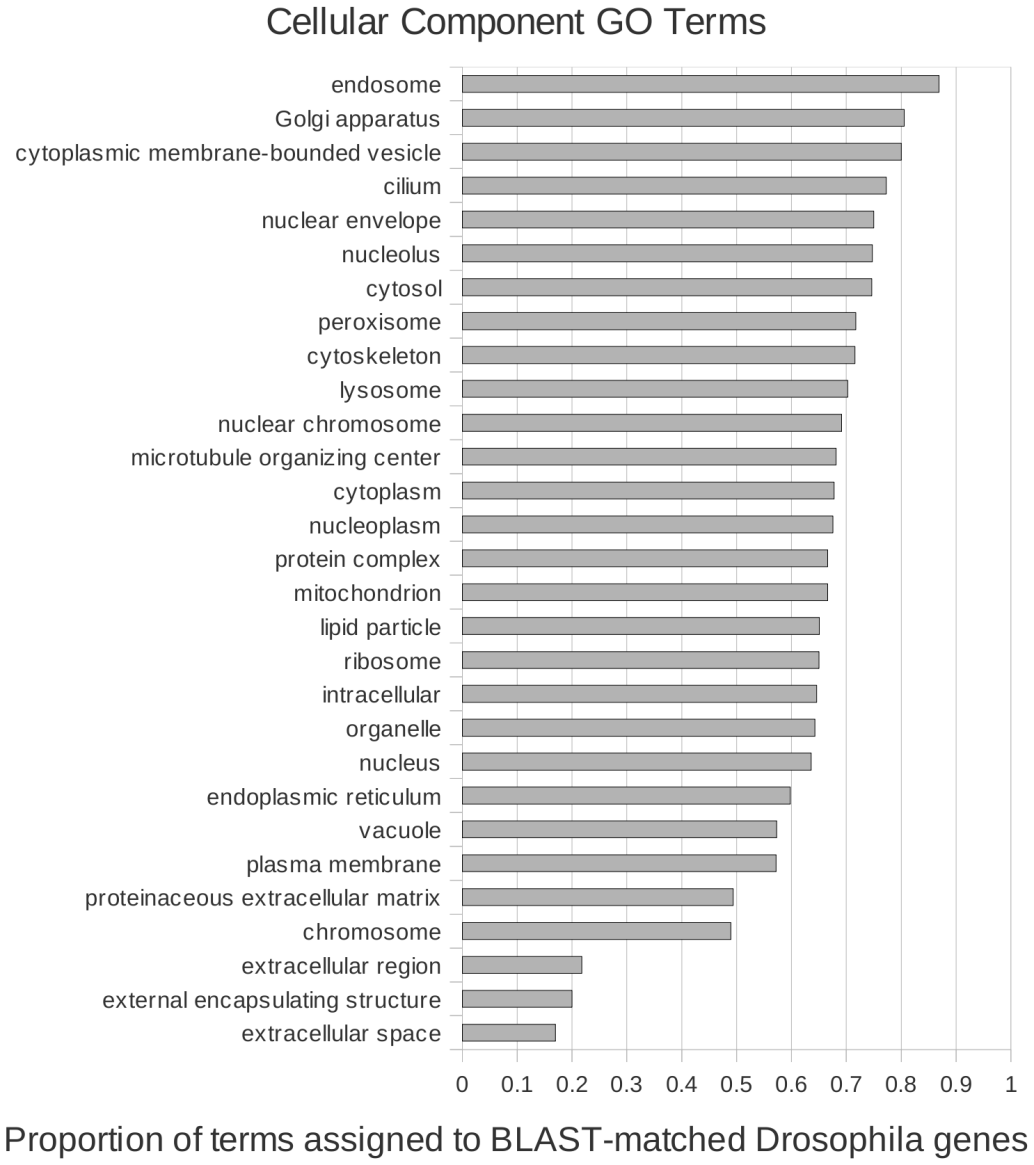
Relative conservation of homology between *Elliptio complanata* and *Drosophila melanogaster* transcriptomes, by cellular component ontology term. The proportion of gene ontology terms assigned to *D. melanogaster* genes that were matched by BLASTX to *E. complanata* contigs, relative to the total number of *D. melanogaster* genes in each GO category.

**Figure 6:**
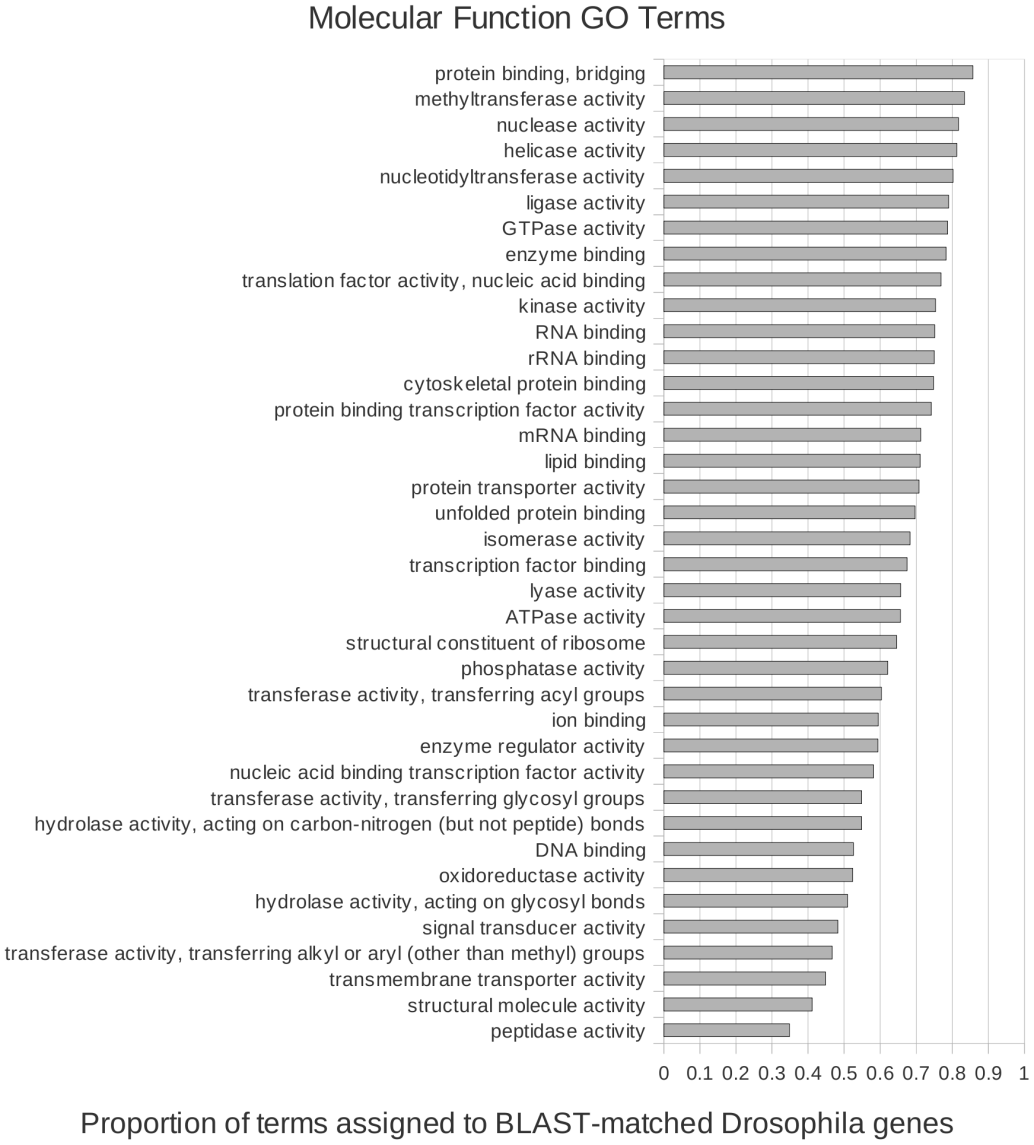
Relative conservation of homology between *Elliptio complanata* and *Drosophila melanogaster* transcriptomes, by molecular function ontology term. The proportion of gene ontology terms assigned to *D. melanogaster* genes that were matched by BLASTX to *E. complanata* contigs, relative to the total number of *D. melanogaster* genes in each GO category.

The relative conservation of genes with GO terms of type ‘cellular component’ and ‘molecular function’ are shown in **Figure 5** and **Figure 6**, respectively; a graph for ‘biological process’ is given in **File S5.**. The most divergent terms for cellular component, e.g. “extracellular space/extracellular region” and “external encapsulating structure”, probably reflect the great divergence in exoskeleton components (cuticle versus shell) between insects and molluscs. Molecular functions “transmembrane transporter activity”, “structural molecule activity”, and “peptidase activity” are among the least conserved of that ontology type. “Reproduction”, “immune system process”, and “carbohydrate metabolic process” are not unexpected representatives of the most divergent biological processes, given the profound developmental and ecological differences between the two species. However, “chromosome organization” and “DNA metabolic process” are comparably divergent. Interestingly, vesicle movement (molecular function: “protein binding, bridging”; cellular component: “endosome”; biological process: “vacuolar transport”) was the most conserved member of all three GO subcategories rather than, for example, genome maintenance or ribogenesis.

### Novel and/or noncoding content of transcriptome

Overall, the large *E. complanata* transcriptome is remarkably poor in protein-level homology to other species. Only 26,689 contigs (19.5%) had protein-level similarity to at least one BLAST database (**Table 2**), with an additional 4,423 having a potential Pfam-A database match at a relaxed expectation (E<0.1). In fact, the highest-coverage contig (Contig_5942) had neither protein or nucleotide homology that could be identified and had no long ORF. Collectively, contigs lacking protein-coding evidence were only modestly shorter on average than those with protein sequence similarity (536.1 bp vs. 561.8 bp, respectively). The longest ORF (between consecutive stop codons) was on average 36.7% of the total length for contigs with sequence similarity to a protein database and 32.3% for those without detected sequence similarity. The paucity of matches to existing protein databases is therefore not simply due to the truncation of protein-coding sequences on short or frame-shifted contigs. However, the 10,000 longest contigs did have a much higher frequency of BLAST matches to at least one database (60.5%), indicating that these contigs are either more likely to be coding or more likely to meet threshold match criteria. Another general explanation for low protein homology is that a large portion of contigs are untranslated regions (UTRs) of protein-coding transcripts, although we are not aware of any previous report of a higher proportion of UTR sequence than coding sequence in shotgun sequencing of randomly primed cDNA. Ultimately, genomic scaffolds and gene models based on full length transcripts are needed to evaluate the proportion of UTR sequence in our assembly.

Alternatively, many transcripts lacking protein-level matches to existing databases may be noncoding RNAs. To evaluate this possibility, we used the program PORTRAIT, a classifier that weights a large number of sequence composition variables that are correlated with coding potential in order to assign a coding probability to a sequence. The distribution of scores for all contigs was bimodal (possible range is 0–1), with a larger number of contigs having low scores, indicating low coding potential, than high scores. However, score was strongly related to contig length, such that almost all low-scoring contigs were shorter than 600 bp, and the large majority of contigs> =  600 bp had high coding probabilities assigned (**File S6**). In contrast, the distribution of PORTRAIT scores for confirmed noncoding RNAs in the NONCODE3 [26] database is unimodal with a very low mean score, regardless of length (**File S7**). Given that the completeness of individual transcript contigs is generally unknown and the lengths of transcript contigs with or without BLAST matches were broadly overlapping, we find the PORTRAIT score distribution to be inconclusive as an overall indicator of how much long, noncoding RNA was captured by our sequencing method (polyA-enriched total RNA). Nonetheless, the longest contigs with low PORTRAIT scores have no credible BLAST matches at either the nucleotide or protein level (e.g., Contig 10, Contig 48, Contig 67, Contig 80, and Contig 92 all exceed 7.5 kb and have scores less than 0.25; see **File S1**), and thus appear to be long non-coding RNA. Direct searches of NONCODE3 with MegaBLAST identified only three potential orthologs in *E. complanata* (at an E-value <1e-8 and with no competing protein match), but these curated noncoding transcripts have been drawn predominantly from a few model organisms and may not be sufficiently conserved in molluscs.

## Conclusions

Our transcriptome assembly for *E. complanata* will support genetic studies of this important indicator species and aid genetic biomarker development. Our results provide additional comparative genomic resources for molluscs, and identify gene families with lineage-specific patterns of diversification. Remarkably, less than a quarter of all contigs had protein similarity above threshold levels, indicating an abudance of noncoding transcripts, untranslated exons, and/or protein-coding sequence that is poorly represented in existing databases.

## Supporting Information

File S1 ***Elliptio complanata*** **annotation summary.** Spreadsheet summarizing annotation data for *E. complanata* transcriptome contigs.
(XLSX)

File S2 **Cytochrome P450 alignment.** Multi-sequence alignment of sequences with cytochrome P450 domains from *E. complanata* and two other molluscs, which is the basis for the gene tree in Figure 2.
(FASTA)

File S3 **Fucosyltransferase alignment.** Multi-sequence alignment of sequences with fucosyltransferase domains from *E. complanata* and two other molluscs, which is the basis for the gene tree in Figure 3.
(FASTA)

File S4 **Galactosyltransferase alignment.** Multi-sequence alignment of sequences with 1,3 beta-galactosyltransferase domains from *E. complanata* and two other molluscs, which is the basis for the gene tree in Figure 4.
(FASTA)

File S5 **Relative conservation of homology between** ***Elliptio complanata*** **and** ***Drosophila melanogaster*** **transcriptomes, by biological process ontology term.** The fraction (horizontal axis) of all gene ontology terms in a given category assigned to *D. melanogaster* genes that were matched by BLASTX to *E. complanata* contigs, relative to the total number of *D. melanogaster* genes in each GO category (category names are on vertical axis). Categories are from the GO-Slim ontology system [15].
(TIF)

File S6 **Histograms of coding-potential scores for** ***Elliptio complanata*** **contigs computed with PORTRAIT.** A. Histogram of coding-potential scores for all contigs in the assembly. B. Histogram of coding-potential scores for contigs greater than 600 bp in length, illustrating a strong length-dependence of coding potential score. Given the unknown completeness of transcript contigs in general, the prevalence of noncoding transcripts in the assembly remains inconclusive but long noncoding transcripts do not appear to be major contributors to the transcriptional complexity in *E. complanata*.
(TIF)

File S7 **Histograms of coding-potential scores computed with PORTRAIT for all NONCODE3 noncoding transcripts, for comparison with PORTRAIT scores computed for** ***Elliptio complanata*** **contigs.** NONCODE3 transcript scores are strongly skewed toward zero regardless of length, in contrast to the pattern observed for *E. complanata* in File S6. High protein-coding potential scores do occur, however.
(TIF)
