# Supplementary figures and images for: Transcriptomic Analysis of the Mussel Elliptio complanata Identifies Candidate Stress-Response Genes and an Abundance of Novel or Noncoding Transcripts
Source: PLoS One. 2014 Nov 6;9(11):e112420. doi: 10.1371/journal.pone.0112420 (PMC4223053; doi:10.1371/journal.pone.0112420)

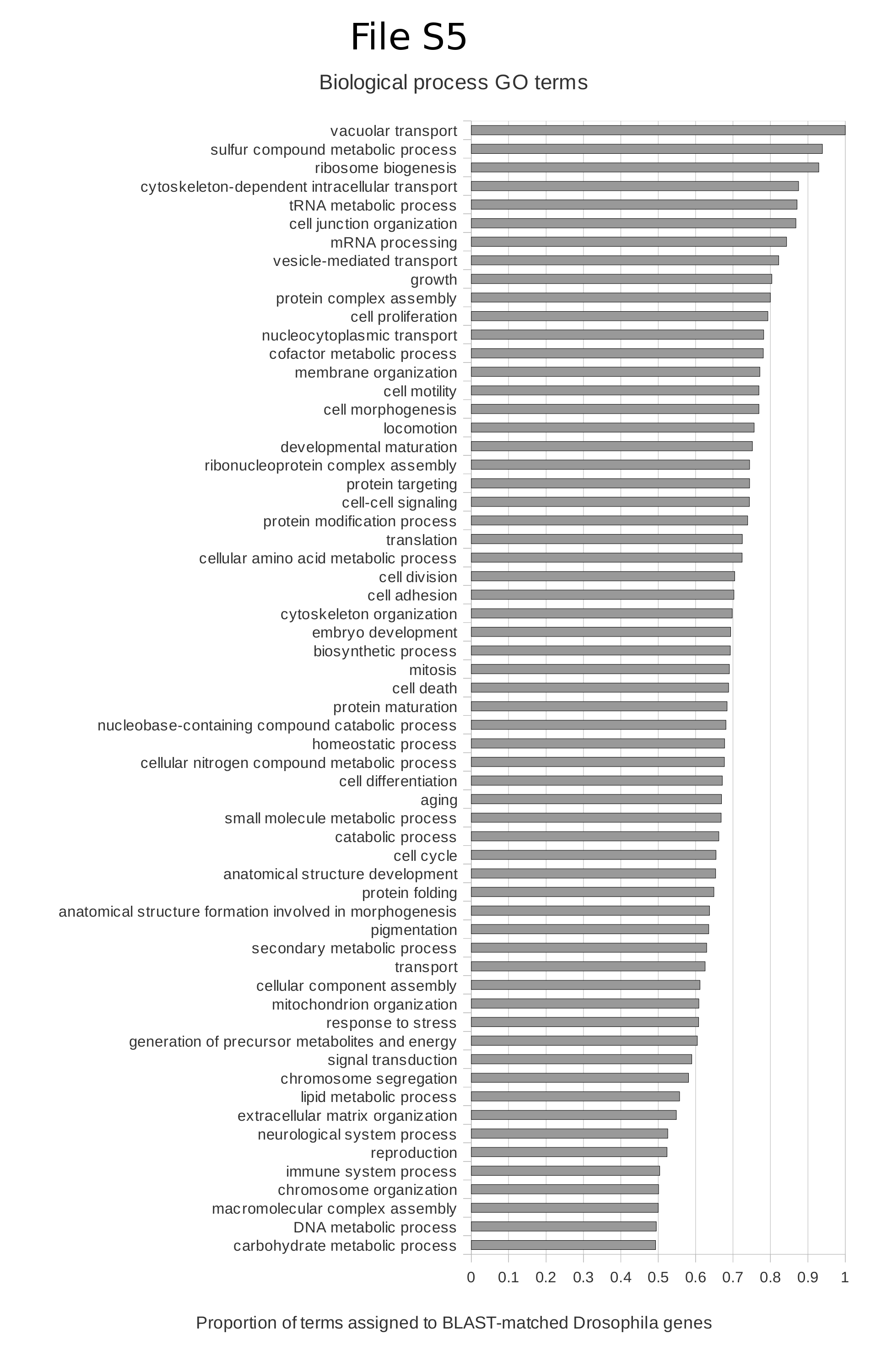

Supplement: File S5 — Relative conservation of homology between Elliptio complanata and Drosophila melanogaster transcriptomes, by biological process ontology term. The fraction (horizontal axis) of all gene ontology terms in a given category assigned to D. melanogaster genes that were matched by BLASTX to E. complanata contigs, relative to the total number of D. melanogaster genes in each GO category (category names are on vertical axis). Categories are from the GO-Slim ontology system [15]. (TIF) [file pone.0112420.s005.tif]

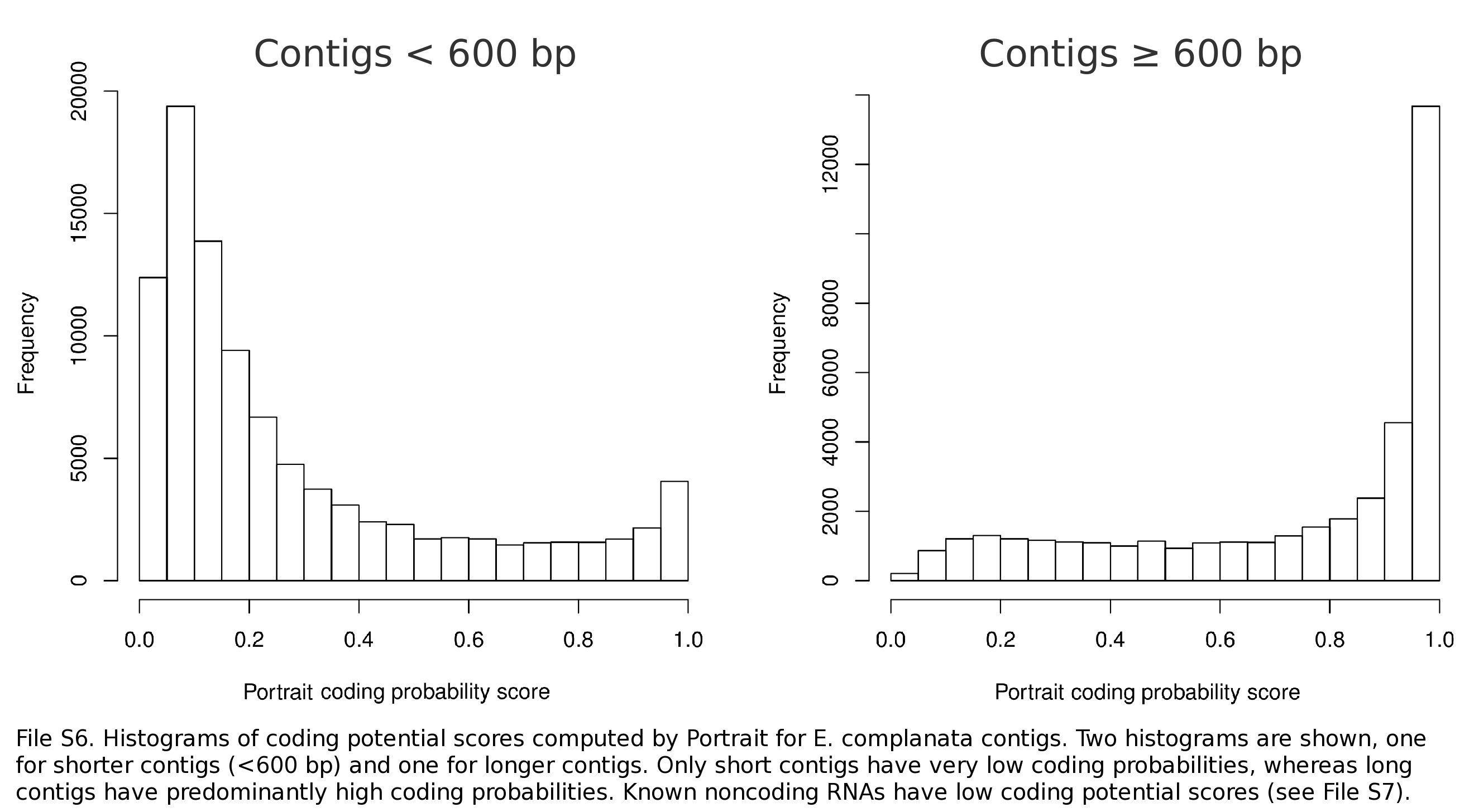

Supplement: File S6 — Histograms of coding-potential scores for Elliptio complanata contigs computed with PORTRAIT. A. Histogram of coding-potential scores for all contigs in the assembly. B. Histogram of coding-potential scores for contigs greater than 600 bp in length, illustrating a strong length-dependence of coding potential score. Given the unknown completeness of transcript contigs in general, the prevalence of noncoding transcripts in the assembly remains inconclusive but long noncoding transcripts do not appear to be major contributors to the transcriptional complexity in E. complanata. (TIF) [file pone.0112420.s006.tif]

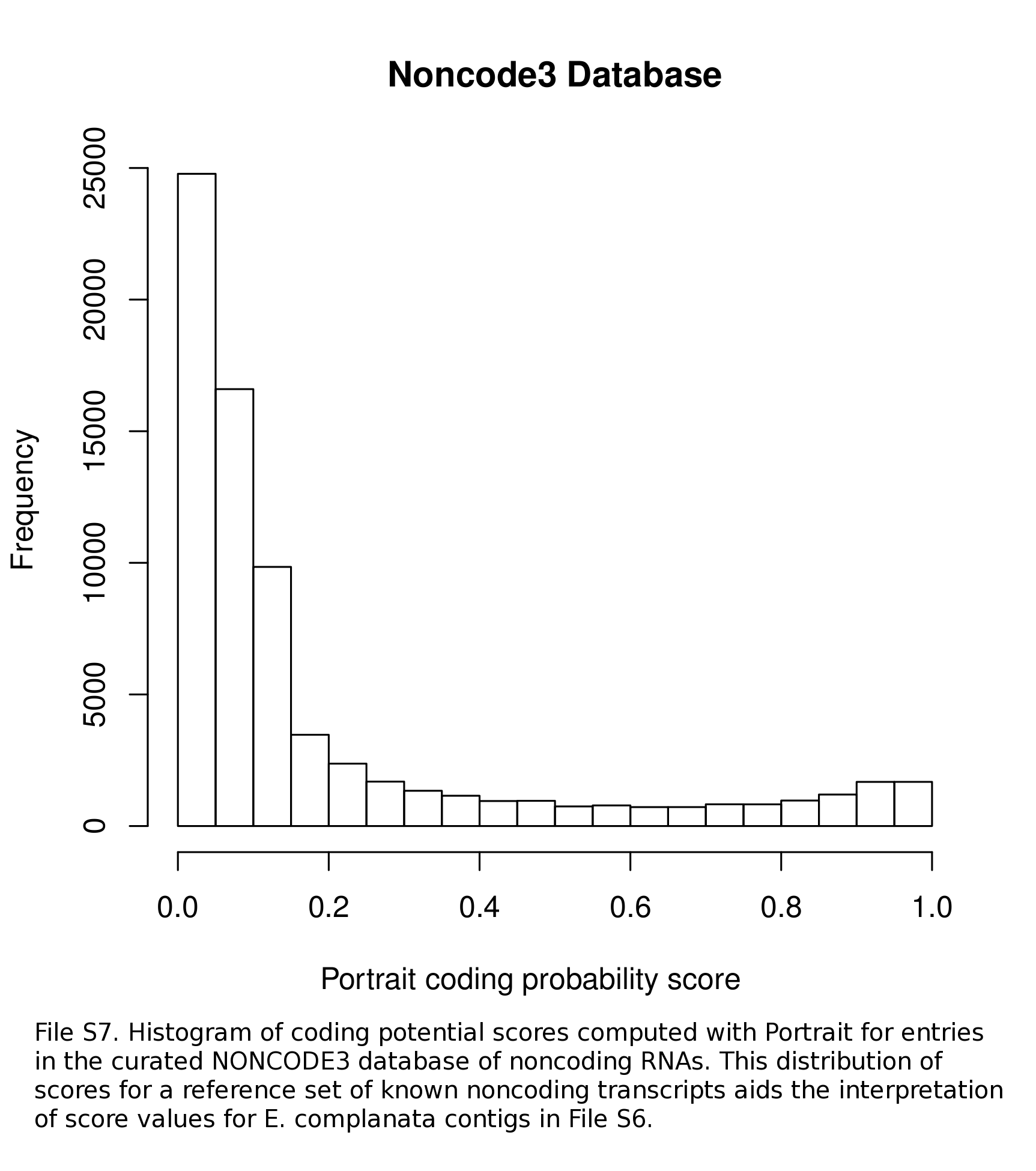

Supplement: File S7 — Histograms of coding-potential scores computed with PORTRAIT for all NONCODE3 noncoding transcripts, for comparison with PORTRAIT scores computed for Elliptio complanata contigs. NONCODE3 transcript scores are strongly skewed toward zero regardless of length, in contrast to the pattern observed for E. complanata in File S6. High protein-coding potential scores do occur, however. (TIF) [file pone.0112420.s007.tif]
